# Generation of Induced Nephron Progenitor-like Cells from Human Urine-Derived Cells

**DOI:** 10.3390/ijms222413449

**Published:** 2021-12-15

**Authors:** Wei-Wei Gao, Jie Zheng, Wonjin Yun, Phil-Jun Kang, Gyuman Park, Gwonhwa Song, In-Yong Kim, Seungkwon You

**Affiliations:** 1 Department of Biotechnology, College of Life Sciences and Biotechnology, Korea University, Seoul 02841, Korea; gaoweiwei@korea.ac.kr (W.-W.G.); devil88128@hotmail.com (J.Z.); nexbolt@korea.ac.kr (W.Y.); rebeast@korea.ac.kr (P.-J.K.); legnaym@korea.ac.kr (G.P.); 2 StemLab, Venture Incubation Center, Korea University, Seoul 136-701, Korea; 3 Institute of Animal Molecular Biotechnology, College of Life Sciences and Biotechnology, Korea University, Seoul 02841, Korea

**Keywords:** nephron progenitor cells, direct reprogramming, transdifferentiation, urine cells, kidney

## Abstract

Background: Regenerative medicine strategies employing nephron progenitor cells (NPCs) are a viable approach that is worthy of substantial consideration as a promising cell source for kidney diseases. However, the generation of induced nephron progenitor-like cells (iNPCs) from human somatic cells remains a major challenge. Here, we describe a novel method for generating NPCs from human urine-derived cells (UCs) that can undergo long-term expansion in a serum-free condition. Results: Here, we generated iNPCs from human urine-derived cells by forced expression of the transcription factors OCT4, SOX2, KLF4, c-MYC, and SLUG, followed by exposure to a cocktail of defined small molecules. These iNPCs resembled human embryonic stem cell-derived NPCs in terms of their morphology, biological characteristics, differentiation potential, and global gene expression and underwent a long-term expansion in serum-free conditions. Conclusion: This study demonstrates that human iNPCs can be readily generated and expanded, which will facilitate their broad applicability in a rapid, efficient, and patient-specific manner, particularly holding the potential as a transplantable cell source for patients with kidney disease.

## 1. Introduction

Chronic kidney disease (CKD) has emerged as a major public health concern due to its prevalence in 7–12% of the population worldwide, progression to irreversible end-stage renal disease (ESRD), impaired quality of life, associations with high social and financial costs, and high rates of associated morbidity and mortality (an 82% increase in CKD epidemic over the past two decades) [1,2]. The current treatment options for kidney failure involve lifelong dialysis and whole kidney transplantation. Although kidney transplantation undoubtedly offers a better quality of life and life expectancy than dialytic treatment, it is limited by the scarcity of available organs and the huge gap between supply and demand. Furthermore, considering that the average life expectancy of dialysis patients is barely a decade, alternative strategies for preventing or delaying the progression to ESRD are urgently needed. In this context, the transplantation of renal stem/progenitor cells is an attractive approach for replenishing damaged renal tissues.

During the past decade, substantial advances have been achieved in differentiating human pluripotent stem cells, including embryonic stem cells (ESCs) and induced pluripotent stem cells (iPSCs), into cells of the kidney lineage [3,4,5]. However, these approaches raise the potential risk of tumor formation and ethical concerns, which restrict their clinical applications [6]. Alternatively, conversion of the fate of somatic donor cells, bypassing the pluripotent stage, provides powerful benefits in terms of karyotypic stability, homogeneity of the target cell population, line-to-line variability, tumorigenic risk, patient-specificity, and time- and labor-efficient processing [7,8]. Recently, intensive efforts in manipulating cell fates employing a set of lineage-specific transcription factors (TFs) and small molecules led to the generation of various cell types, including neurons [9], oligodendrocyte precursor cells [10], cardiomyocytes [11], and hepatocyte-like cells [12]. Furthermore, there is emerging interest in lineage conversion using pluripotency factors [13,14,15]. This strategy focuses on driving cells toward an epigenetically unstable/plastic state during reprogramming, which means they can be directed toward an alternative cell fate rather than toward iPSCs when exposed to proper signaling environments [16]. Despite these impressive exhibitions of phenotypic conversion, a few studies have reported TF-driven transdifferentiation into cells of the kidney lineage to the best of our knowledge. Hendry et al. defined six TFs (SIX1, SIX2, OSR1, EYA1, HOXA11, and SNAI2 (referred to as SLUG)) that are capable of inducing dedifferentiation of HK2 cells, an immortalized proximal tubular cell line, into a precursor state in combination with valproic acid [17]. Afterward, they attempted to generate nephron progenitor-like cells from HK2 cells using an inducible transposon system with three TFs (SNAI2, EYA1, and SIX1) [18], however, these two approaches are unsuitable for patient-specific clinical trials due to the poor accessibility of these primary epithelial cells.

In this study, we generated iNPCs from human urine-derived cells (UCs), obtained from healthy male and female donors, by forced expression of the TFs OCT4, SOX2, KLF4, and c-MYC (OSKM, also known as Yamanaka factors) together with SLUG. This process occurred via modulation of the BMP, FGF, and WNT signaling pathways, providing cues for self-renewal and lineage commitment of NPCs [4]. In addition to canonical TFs that orchestrate the reprogramming process, SLUG was selected as an early epithelial-to-mesenchymal transition (EMT) regulator to promote reprogramming. ESC-derived NPCs (referred to as ESC-NPCs) served as a control, which is used as a golden standard for in vitro pluripotency [6,19]. The generated iNPCs resembled human ESC-NPCs in terms of their morphology, biological characteristics, in vitro differentiation capacities, and global gene expression.

## 2. Results

### 2.1. Screening for NPC-Inducing Factors

Based on previous evidence [17,20] and our preliminary results in combination with renal-specific TFs, we selected five TFs that potentially participate in inducing the NPC phenotype (OSR1, SIX1, SIX2, and PAX2) or promote reprogramming as an EMT regulator (SLUG) for screening NPC-inducting TFs. To convert to an NPC phenotype, we retrovirally transduced human UCs at early passages 2–5 with these factors and then cultured these cells in a chemically defined medium containing FGF2, FGF9, activin A, and retinoic acid for 9–15 days, which was based on the study that reported the renal lineage differentiation of human ESCs and iPSCs [21]. At day 15 post-induction, few putative iNPC-like colonies were observed, but more importantly little or no expression of endogenous SIX2 (Figure S1a), widely regarded as one of the critical makers for nephron progenitors, was detected while expanding the picked colonies. Afterward, Yamanaka factors were examined for generating NPCs because their transient overexpression has been well-established to change cell fates between developmentally distant cell types [22]. We attempted to generate three putative NPC lines from UCs overexpressing OSKM. mRNA level expression of typical nephron progenitor makers such as SIX2, CITED1, EYA1, WT1, and GDNF was detected in these cells (Figure S1b). Although expression of the pluripotency markers OCT4 and NANOG were lower in OSKM-transduced UCs than in iPSCs [23], their expression exhibited considerable levels in acquiring a pluripotent status. Based on these results, we investigated whether OSKM in combination with SLUG (referred to as OSKM-SLUG), enabling to regulate EMT, has a synergistic effect on the generation of iNPCs from UCs and allows these cells to maintain their phenotype. Surprisingly, expression of SIX2 and CITED1, which are cap mesenchyme (CM) markers, was elevated in OSKM-SLUG-transduced UCs, while expression of pluripotency markers (NANOG and OCT4) was reduced (Figure S1c). These findings indicate that the generated iNPCs have different pluripotent behaviors from iPSCs, but are similar to ESC-NPCs. ESC-NPCs served as a positive control (Figure S2a,b), previously well-established [24]. Although the combination of OSKM and SLUG exhibited the highest similarity to ESC-NPCs in the phenotype, these cells were not expandable under the present culture condition and gradually lost their progenitor properties (data not shown).

### 2.2. Generation of iNPCs from Human UCs

**Figure 1:**
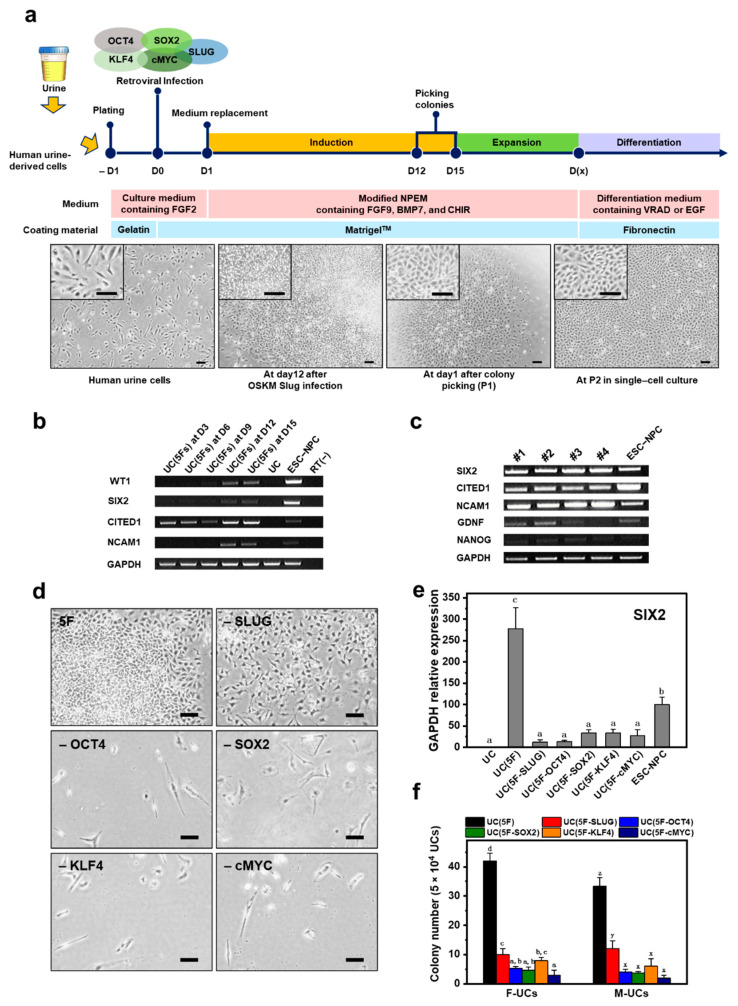
Generation of iNPCs from human UCs. (**a**) Schematic for generation of iNPCs from human UCs and renal differentiation. The images show morphological changes of UCs during the conversion process. At 12 days post-induction, the colonies were picked for further expansion and characterization. Scale bar = 200 and 100 μm for the insert images; (**b**) RT–PCR analysis of NPC-specific markers (WT1, SIX2, CITED1, and NCAM1) as a function of time; (**c**) RT-PCR analysis of NPC-specific (SIX2, CITED1, GDNF, and NCAM) and pluripotency (NANOG) markers in the generated four iNPC lines; (**d**) Morphologies of UCs infected with 5F, 5F-SLUG, 5F-OCT4, 5F-SOX2, 5F-KLF4, and 5F-cMYC at 12 days of induction. Scale bar = 200 μm; (**e**) Relative mRNA expression of SIX2 as a strong indicator of successful generation of NPCs; (**f**) Number of colonies formed by the cells infected with 5F, 5F-SLUG, 5F-OCT4, 5F-SOX2, 5F-KLF4, and 5F-cMYC at 12 days post-induction. Cells were seeded at a density of 5 × 10^4^ cells per well in a 6-well plate. F- and M-UCs indicate female- and male-derived UCs, respectively. Data are represented as mean ± SD. Different letters indicate significant differences between groups (*p* < 0.05).

Figure 1a outlines the process by which functional NPCs were successfully generated from human UCs. To maintain and expand undifferentiated NPCs, OSKM-SLUG-transduced UCs were immediately exposed to the nephron progenitor niche, which contained FGF9, Heparin, BMP7, LDN-193189, CHIR99021, and Y-27632. This signaling environment was established by modifying nephron progenitor expansion medium (mNPEM) as previously described [4], in which SIX2+/CITED1+ CM progenitors derived from embryonic kidneys or human ESCs can be propagated by manipulating the BMP, FGF, and WNT signaling pathways while preserving the potential for renal differentiation. At 12 days post-transfection, colonies were observed and expanded as single cells on Matrigel-coated plates by manual picking (Passage 1). Upon further passaging, putative NPCs with a small, spindle-shaped mesenchymal morphology similar to that of ESC-NPCs were established (Figure 1a and Figure S2a). During the conversion process, expression of the NPC markers WT1, SIX2, CITED1, and NCAM1 increased in these cells, comparable to ESC-NPCs (Figure 1b) [25]. Moreover, mRNA expression of pluripotency (NANOG) and NPC (SIX2, CITED1, GDNF, and NCAM1) markers were analyzed in four established lines of iNPCs (Figure 1c). Specification of posterior intermediate mesoderm into NPCs of the metanephric mesenchyme (MM) is characterized by co-expression of WT1, SALL1, PAX2, and GDNF [26,27]. The reciprocal interaction between the MM and ureteric bud tips contributes to the formation of dense clusters called the CM in which SIX2 and CITED1 expression is activated [28,29]. Accordingly, up-regulation of endogenous SIX2 and CITED1 is considered to be a strong indicator of successful conversion into NPCs [30]. Meanwhile, NCAM1+ cells, found in human fetal kidneys, retain their nephrogenic potential during in vitro culture and elicit beneficial effects on the progression of kidney disease [31]. NCAM1 is downregulated during differentiation into kidney epithelial cells and re-activated in a specific subset of cells that undergo dedifferentiation to behave as highly stem/progenitor cells (e.g., in the regenerative response following kidney damage) [32]. Furthermore, GDNF signaling through the Ret receptor is required for ureteric bud growth and branching morphogenesis during kidney development [33].

In an attempt to determine the minimally required set of TFs for the generation of iNPCs from human UCs, individual factors were removed from the pool of OSKM-SLUG. Conversion of human UCs into iNPCs involved EMT-like morphologic changes and mRNA expression of SIX2 and CITED1 (Figure 1d,e and Figure S1d). mRNA expression of SIX2 was markedly reduced by removal of each of the five TFs and was highest in cells co-overexpressing all five TFs, rather than in ESC-NPCs. These findings were consistent with the iNPC colony-forming efficiencies of female- and male-derived UCs (referred to as F- and M-UCs, respectively) (Figure 1f).

### 2.3. Characterization and In Vitro Expansion of iNPCs

**Figure 2:**
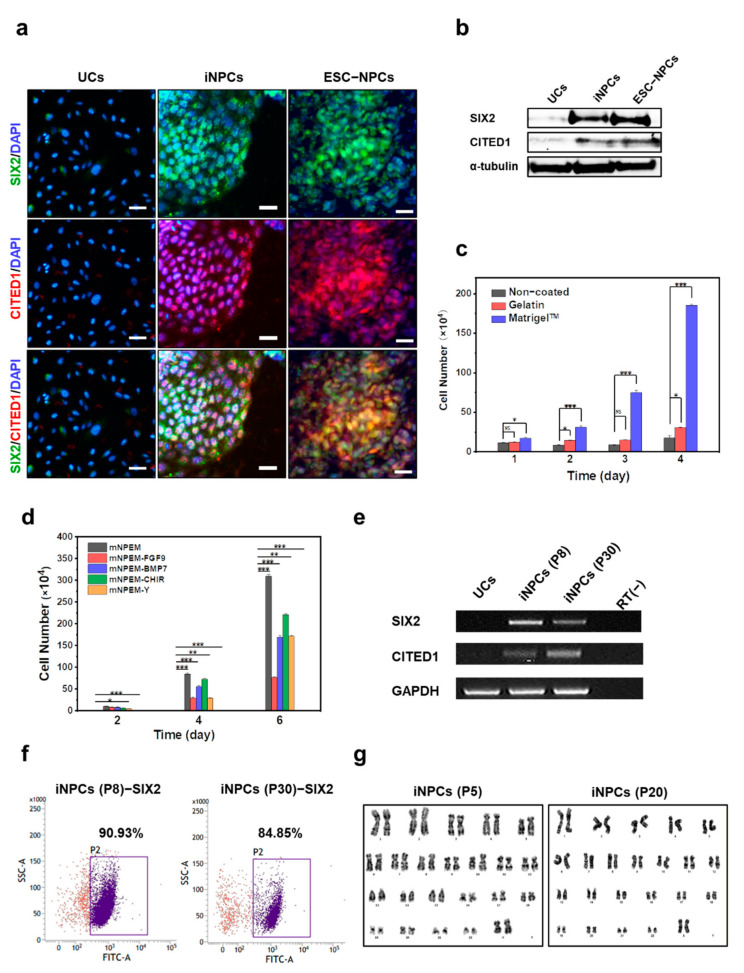
Characterization and in vitro expansion of iNPCs. (**a**) Immunofluorescence analysis of NPC-specific markers SIX2 (green) and CITED1 (red) in UCs, iNPCs, and ESC-NPCs. Scale bar = 100 μm, Nuclei were counterstained with DAPI; (**b**) Western blot analysis of NPC markers SIX2 and CITED in UCs, iNPCs, and ESC-NPCs; (**c**,**d**) Optimization of culture conditions for in vitro expansion of iNPCs; (**c**) Cell proliferation of iNPCs in non-, gelatin- and Matrigel-coated plates in mNPEM for 4 days. Cells were seeded at a density of 5 × 10^4^ cells per well in a 6-well plate. N.S., no significant difference; (**d**) Cell proliferation at day 6 of iNPCs in Matrigel-coated plates in the medium supplemented with FGF9, BMP7, CHIR99021, and Y-27632, in combination or as individually removed. Cells were seeded at a density of 5 × 10^4^ cells per well in a 6-well plate. Data are represented as mean ± SD. * denotes a statistically significant difference with * *p* < 0.05, ** *p* < 0.01, *** *p* < 0.001. Scale bar = 100 μm; (**e**) RT-PCR analysis of NPC marker SIX2 and CITED1 in early- and late-passage iNPCs (P8 and 30, respectively); (**f**) Flow cytometry data showing expression of SIX2 as a function of passage number. Data are representative of three independent experiments; (**g**) Karyotyping (G-banded) of iNPCs derived from female human UCs at passages 5 and 20.

The iNPCs established from F-UCs (Figure 2a,b) expressed the CM markers SIX2 and CITED1, consistent with the immunostaining and western blot results obtained with ESC-NPCs. ESC-NPCs served as a positive control. The exogenous expression of each of the reprogramming genes OSKM and EMT regulator SLUG was evaluated by RT-PCR, which demonstrated that all five genes (OSKM and SLUG) were expressed in iNPCs at passage 8 (Figure S3a). By contrast, except for endogenous SLUG, there was only minimal or no expression of the endogenous OSKM genes in the iNPCs (P8) compared with human iPSCs-positive control (Figure S3b). We next injected these cells into immunodeficient mice and monitored tumorigenesis over 12 weeks (Figure S3c); no tumor formation was observed in comparison with the U87MG cell- and iPSC-injected sites, implying the absence of tumorigenic potential of iNPCs.

In vitro expansion of iNPCs is an important prerequisite for potential medical applications, including cell therapy, renal disease modeling, drug screening, and reconstitution of the functional kidney in vitro. Related studies proposed that an in vitro nephron progenitor niche can expand NPCs derived from embryonic kidneys or human ESCs by modulating the FGF, BMP, and WNT pathways [4,5]. Based on previous evidence, we optimized serum-free conditions for the propagation of iNPCs generated from UCs. To investigate the effect of the coating material on cell growth, we compared the proliferation of iNPCs on non-, gelatin- and Matrigel-coated plates (Figure 2c and Figure S4a), which are widely used for stem cell culture, in modified NPEM was evaluated by removing each one from mNPEM (Figure 2d and Figure S4b). Removal of each additive reduced cell proliferation, while FGF9 was a key supplement for in vitro expansion of these cells. The established iNPCs were propagated in mNPEM on Matrigel-coated plates for 30 passages, with repeated freeze-thaw cycles, and continued to robustly express the CM markers SIX2 and CITED1; SIX2+ population purity was 90.93% and 84.85% at passages 8 and 30, respectively (Figure 2e,f). iNPCs generated from F- and M-UCs retained a normal karyotype for at least 20 (Figure 2g) and 10 passages (Figure S4c), respectively, demonstrating their stable expandability in vitro.

### 2.4. Global Gene Expression Analysis of iNPCs

**Figure 3:**
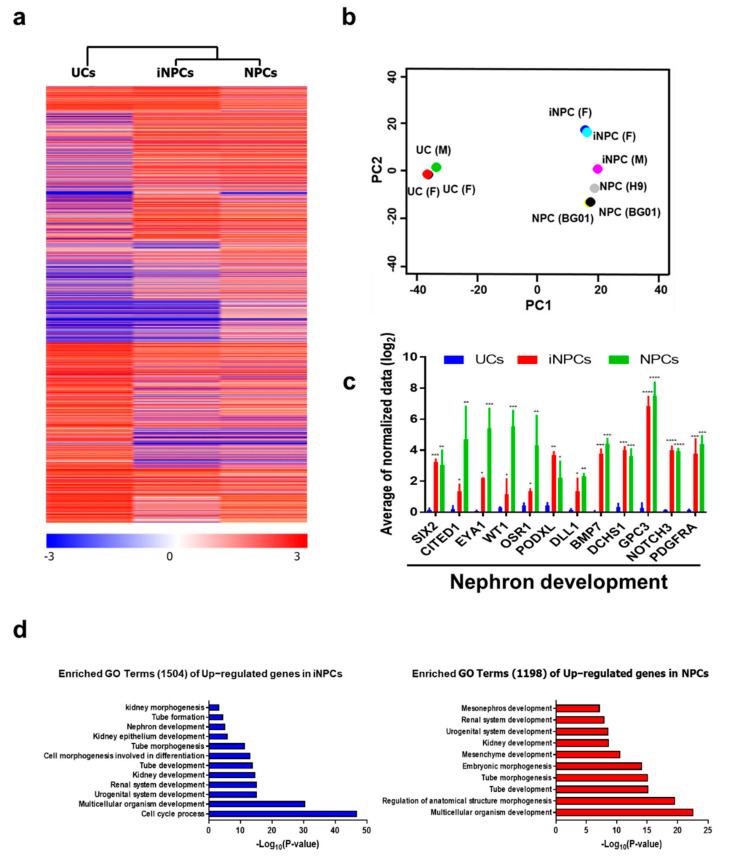
Global gene expression analysis of iNPCs. (**a**) Heatmap for Hierarchical clustering of 26,256 genes differentially regulated between UCs, iNPCs, and NPCs (referred to ESC-NPCs), as determined by RNA-seq. Red and blue in the heat map indicate upregulated and downregulated genes, respectively; (**b**) Principal component analysis (PCA) of RNA-seq data from UCs (2 F- and 1 M-UCs), iNPCs (2 F-UC and 1 M-UC-iNPCs), and NPCs (1 H9-ESC- and 2 BG01-ESC-NPCs); (**c**) Comparative expression of nephron development-related genes in UCs, iNPCs, and NPCs; (**d**) GO analysis of overlapping upregulated genes in iNPCs vs. UCs (left) and BG01-NPCs vs. UCs (right). Data shown reflect mean expression levels from cell lines and biological replicates belonging to each cell group. Data are represented as mean ± SD. * denotes a statistically significant difference with * *p* < 0.01, ** *p* < 0.01, *** *p* < 0.001, **** *p* < 0.001.

Next, we compared the global gene expression patterns of iNPCs with those of parental UCs and ESC-NPCs by RNA sequencing (RNA-seq). Hierarchical clustering of the whole transcriptome (26,256 genes) showed the genome-wide conversion of F- and M-UCs into iNPCs and demonstrated a high degree of similarity between ESC-NPCs and iNPCs (Figure 3a). In particular, the scatter plot of the first two principal components revealed a close relationship between iNPCs (3 independent F- and M-iNPC lines) and ESC-NPCs (3 independent BG01-ESC- and H9-ESC-NPC lines), while clearly demonstrating the successful separation of iNPCs and UCs (Figure 3b). While UCs exhibited no or low expression in a gene set related to nephron development (SIX2, CITED1, EYA1, WT1, OSR1, PODXL, DLL1, BMP7, DCHS1, GPC3, NOTCH3, and PDGFRA), these genes were highly upregulated in both iNPCs and ESC-NPCs (Figure 3c), which is supported by the qPCR analysis in SIX2, CITED1, EYA1, WT1 and OSR1 (Figure S5). These comparisons indicate that UCs were converted into iNPCs with renal progenitor characteristics comparable to ESC-NPCs. Furthermore, similar to ESC-NPCs, the gene ontology (GO) categories significantly enriched in iNPCs were gene sets related to kidney development and nephrogenesis (Figure 3d). These results suggest that the generated iNPCs were almost entirely converted to NPC fate during the conversion process and thus possessed lineage specificity at the global RNA level.

### 2.5. Differentiation Potential of iNPCs

**Figure 4:**
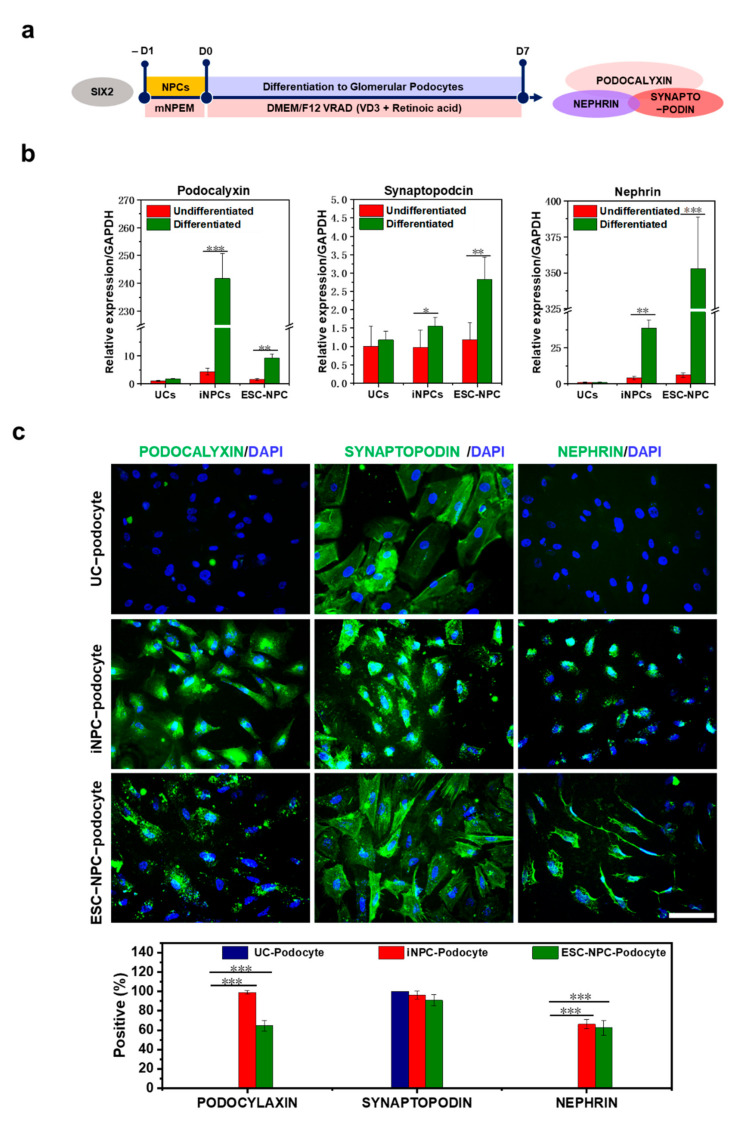

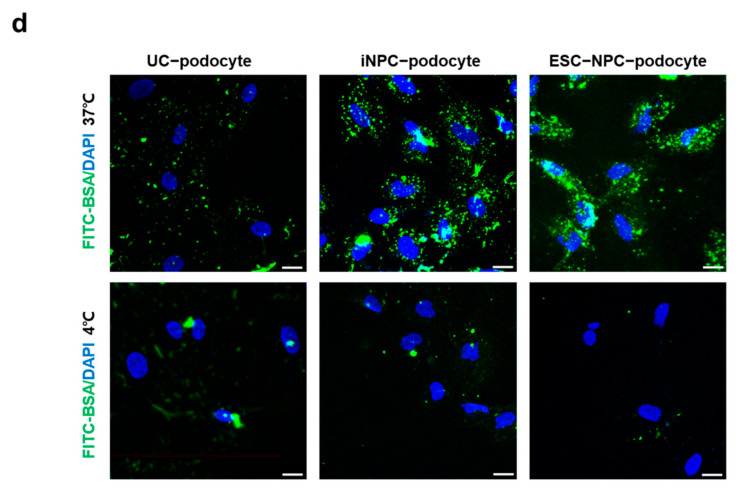
Differentiation of iNPCs into podocyte and functional analysis. (**a**) Schematic representation of protocol for differentiation of iNPCs into podocytes. iNPCs were cultured with VRAD medium for 7 days; (**b**) qRT-PCR analysis for podocyte-specific markers nephrin, synaptopodin, and podocalyxin in UCs, iNPCs, and ESC-NPCs before or after podocyte differentiation. The expression levels were normalized to GAPDH; (**c**) Immunofluorescence and quantitative analysis of podocyte-specific markers podocalyxin (green), SYNAPTOPODIN (green) and NEPHRIN (green) in iNPC-derived podocyte, Scale bar = 100 μm; (**d**) Albumin uptake by iNPC-derived podocytes at day 7 of differentiation. FITC- albumin is shown in intracellular vesicles. UCs and ESC-NPCs served as a cell source for negative and positive controls, respectively. Nuclei were counterstained with DAPI, Scale bar = 20 μm. Data are represented as mean ± SD. * denotes a statistically significant difference with * *p* < 0.05, ** *p* < 0.01, *** *p* < 0.001.

**Figure 5:**
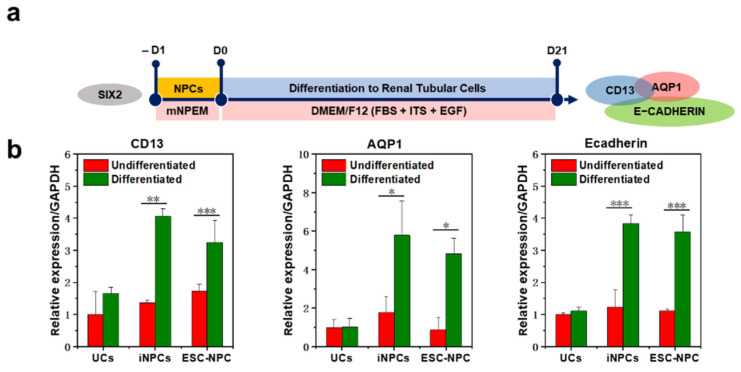

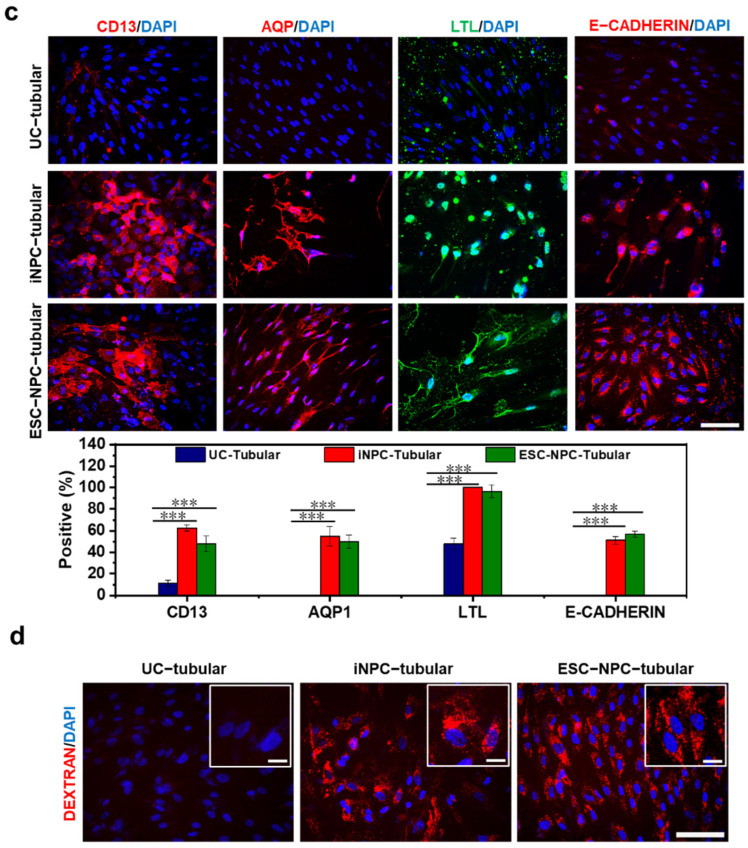
Differentiation of iNPCs into renal tubular cells and functional analysis. (**a**) The schematic diagram for differentiation of iNPCs into renal tubular cells in vitro; (**b**) qRT-PCR analysis for proximal tubular cells markers tubular cell-specific markers CD13, AQP1 and E-CADHERIN in UCs, iNPCs, and ESC-NPCs before or after tubular differentiation. The expression levels were normalized to GAPDH; (**c**) Immunofluorescence and quantitative analysis of renal tubular cell-specific markers CD13 (green), AQP1 (red), LTL (green), and E-CADHERIN (red) in UCs, iNPCs, and ESC-NPCs after tubular differentiation; (**d**) Functional analysis of iNPC-derived tubular cells by dextran uptake. The day-21 differentiated cells were exposed to 10 μg/mL Alexa Fluor 555 dextran at 37 °C for 24 h. UCs and ESC-NPCs served as a cell source for negative and positive controls, respectively Nuclei were counterstained with DAPI. Scale bar = 100 μm, insert scale bar = 20 μm. Data are represented as mean ± SD. * denotes a statistically significant difference with * *p* < 0.05, ** *p* < 0.01, *** *p* < 0.001.

**Figure 6:**
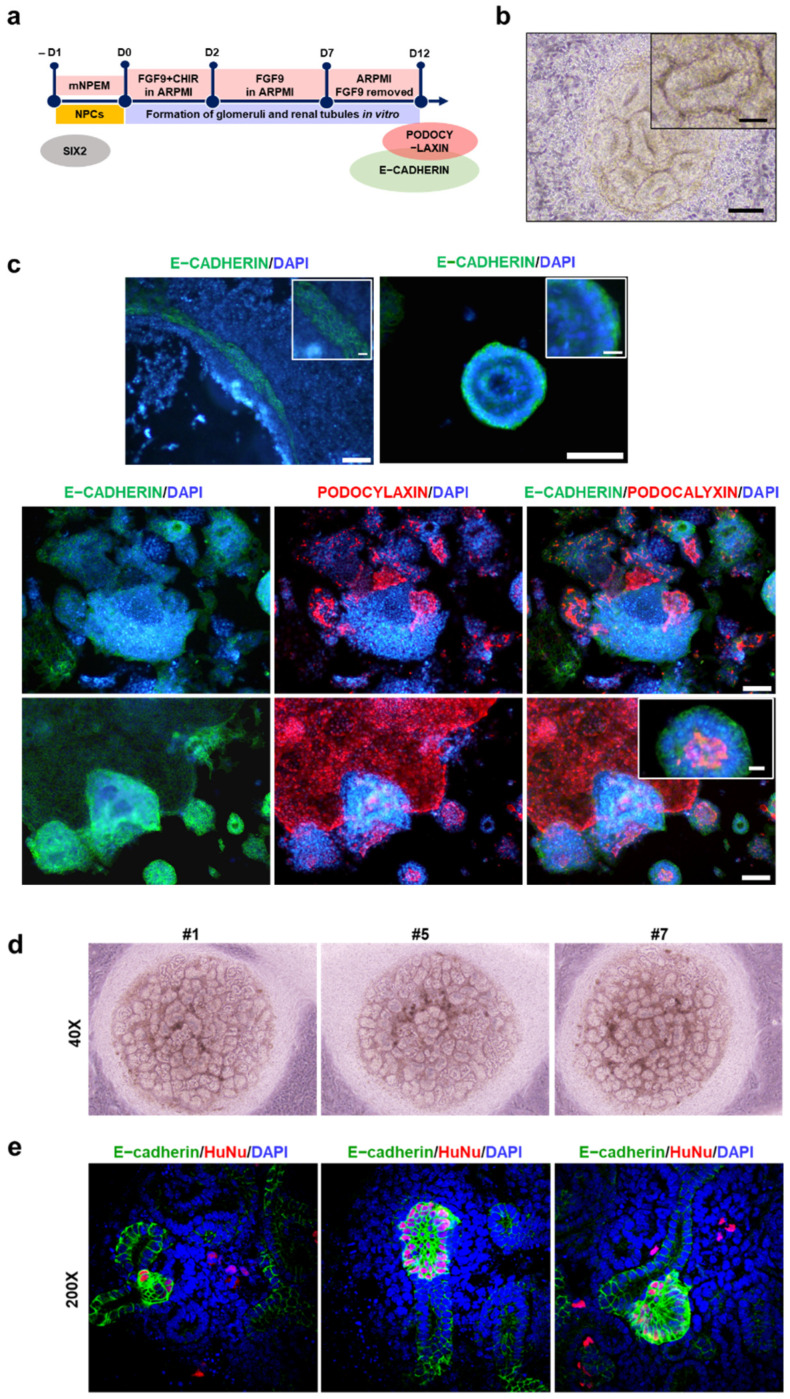

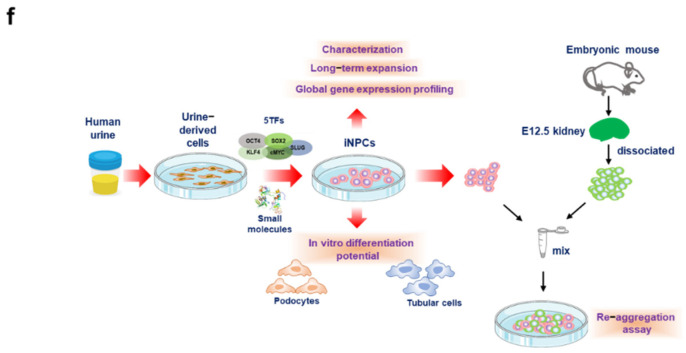
The nephrogenic aggregate potential of iNPCs in vitro. (**a**) Schematic representation for the stepwise formation of glomeruli and renal tubules from iNPCs in vitro; (**b**) Representative phase-contrast image of aggregated renal tubular cells generated from iNPCs in the early stage of differentiation; (**c**) Immunofluorescence analysis of expression of the distal tubular cell (E-CADHERIN, green) and glomerular podocyte (PODOCYLAXIN, red)-specific markers. Nuclei were counterstained with DAPI. Scale bar = 100 and 20 μm for the insert images; (**d**,**e**) Reaggregation of iNPCs and mouse embryonic kidney cells; (**d**) Optical images showing the morphology of 7-day chimeric aggregate culture of iNPCs and E12.5 mouse embryonic kidney cells on a filter at the air–medium interface; (**e**) Immunofluorescent staining of HuNu+ and E-cadherin (distal tubular marker) in day-7 aggregate culture; (**f**) Schematic describing the generation of iNPCs from human urine-derived cells.

To verify that the established iNPCs could differentiate into the main components of the nephron, human female UC-derived iNPCs were transferred to fibronectin-coated plates in mNPEM and 1 day later exposed to previously reported media with specific compositions for inducing differentiation into glomerular podocytes or renal tubular cells (Figure 4a and Figure 5a) [34,35]. F-UCs and ESC-NPCs served as negative and positive controls, respectively. After 1 week of differentiation, we observed a homogeneous population of multinucleated cells with a large cell body and an arborized morphology (Figure S6a), which is consistent with a previous study showing podocytes generated from human iPSCs [36]. qRT-PCR analysis indicated that expression of podocyte-specific markers, such as podocalyxin, synaptopodin, and nephrin, was significantly increased in the podocytes differentiated from iNPCs and ESC-NPCs when compared to UCs cultured under the same condition (Figure 4b). Meanwhile, the expression of SIX2 in iNPCs and ESC-NPCs was decreased during podocyte differentiation (Figure S6b). These results are in agreement with the previous finding that SIX2+ NPCs gradually give rise to nephron epithelia in which cells exhibit downregulation of SIX2 and express renal subtype-specific markers [21,37]. Moreover, immunostaining analysis demonstrated that iNPC- differentiated podocytes expressed the podocyte-specific protein SYNAPTOPODIN, PODOCYLAXIN, and NEPHRIN, which was similar to ESC-differentiated podocytes (Figure 4c and Figure S6c). Functional activity of the differentiated podocytes was determined by endocytic uptake of FITC-labeled albumin at 4 °C (inhibits albumin endocytosis) or 37 °C (permits endocytosis) [38,39]. Podocytes play a key role in the glomerular filtration barrier that impedes the passage of large proteins and macromolecules such as albumin from the blood to the urinary ultrafiltrate. As shown in Figure 4d and Figure S6d, albumin-containing vesicles were observed within the podocytes placed at 37 °C, but very low at 4 °C. There was a significant increase in the amount of albumin taken up by iNPC- and ESC-NPC-differentiated podocytes compared to the cells derived from UCs. Next, differentiation of iNPC lines into renal tubular cells was observed after 3 weeks of induction, with mesenchymal-to-epithelial morphological changes. These cells expressed the proximal tubular cell-specific markers CD13, AQP1, and LTL and epithelial maker E-CADHERIN, as determined by qRT-PCR and immunostaining (Figure 5b,c). Moreover, the differentiated cells showed the functional activity of the proximal tubule via uptake of fluorescently labeled dextran as previously reported [40]. While a substantial amount of dextran was accumulated in iNPC- and ESC-differentiated tubular cells, very little dextran was found in the cells induced from UCs (Figure 5d and Figure S6e). Thus, these results demonstrate the differentiation potential of iNPCs in directed differentiation towards functional podocytes and renal tubular cells. Human ESC and iPSC-derived NPCs undergo the mesenchymal-to-epithelial transition (MET) and form glomeruli and renal tubules when exposed to FGF9 and a low dose CHIR (Figure 6a) [37]. Similarly, the generated iNPCs and ESC-NPCs were aggregated and underwent nephrogenesis (Figure 6b,c and Figure S7). These clonally derived aggregates expressed segmental markers of the nephron, including glomerular podocytes (PODOCYLAXIN) and renal tubules (E-CADHERIN), and had lumens (Figure 6c). These observations are consistent with earlier findings concerning nephron structures induced from ESCs and iPSCs [5,21,41]. In the human kidney, E-CADHERIN is abundant in the distal tubule, while PODOCYLAXIN is more dominant in the glomeruli (E-CADHERIN+/PODOCYLAXIN) [37,42]. Interestingly, the immunostaining images reveal the expression of these two markers, indicating the co-existence of immediate precursors of distal tubule epithelial cells and podocytes. These results imply the potential for generating a kidney organoid originated from the immature cell clusters consisting of multiple cell types, including podocytes and tubular cells. To further support the nephrogenic potential of UC-derived iNPCs, we attempted to perform a chimeric aggregate assay by mixed culture with E12.5 mouse embryonic kidney cells at the liquid-air interface for 7 days, as previously described [43,44,45]. The chimeric aggregates were dynamically changed to the morphology of tubular branches and renal vesicles, similar to the complex tubular epithelial networks (Figure 6d). The converted cells (HuNu+) were found to integrate into E-cadherin+ nephron segments (Figure 6e). Nevertheless, the chimeric aggregates were maintained for up to 7 days and after then, they were disintegrated, which is similar to what was observed for chimeric aggregate analysis with iPSC-derived kidney progenitors [18,45].

## 3. Discussion

Despite substantial progress in understanding the embryologic origin of mature cell types in the adult kidney, accumulated evidence indicates that the kidney is unable to generate new nephrons in response to injury. Indeed, renal progenitors disappear prior to birth, possibly because their niche is lost [46]. The adult kidney has a robust capacity to undergo epithelial turnover via injury-induced dedifferentiation of terminally differentiated epithelia or via these cells transiently acquiring the phenotype of progenitors with reparative properties [47,48]. Increased understanding of the repair mechanisms of the kidney and the limited renal recovery has encouraged researchers to investigate the functionality of other cells in the setting of kidney diseases. Most studies have focused on reprogramming of human ESCs and iPSCs into kidney lineage cells, despite the risk of tumor formation and ethical concerns [3,21]. Here, we described the generation of expandable iNPCs from human UCs by forced expression of OSKM-SLUG, followed by exposure to a cocktail of defined small molecules. In addition to OSKM, which orchestrates the reprogramming process, SLUG was selected as an early EMT regulator to promote reprogramming (Figure 1 and Figure S1). During embryogenesis and development, switching between epithelial and mesenchymal states in an embryo contributes to developmental processes such as nephrogenesis [49,50,51]. Indeed, the EMT master regulator SLUG is found in the MM renal stage progenitors during kidney development, and more interestingly, renal tubular epithelial cells in the regenerative response to injury undergo EMT-mediated dedifferentiation to a more primitive stage, allowing them to re-proliferate, re-differentiate, and repair the nephron [52,53]. Pluripotency-factor-driven lineage conversion relies on the re-activation of epigenetically repressed lineage-specific genes and, at an early stage of reprogramming, guiding cell fates toward a lineage-specific path rather than toward the iPSC fate by cell activation and signaling-directed lineage transition [16]. Recent studies provided important insights into the responses of NPCs derived from embryonic kidneys or derived from human ESCs to modulation of the FGF, BMP, and WNT signaling pathways (Figure 2), which regulate the balance between renewal and differentiation of these cells [4,5]. In the present study, the generated iNPCs were similar to ESC-NPCs in terms of their morphology, biological characteristics, differentiation potential, and global gene expression (Figure 6f).

Our approach has advantages over other cell sources for clinical applications. First, the present method manipulates the fate of human UCs, allowing personalized medicine via a non-invasive method. Moreover, considering the origin of UCs, it may be the most suitable cell source for generating patient-specific NPCs. Second, the generation of a functional cell type into another lineage is a promising tool for extensive clinical trials, minimizes the potential risk of tumorigenesis after transplantation by bypassing the pluripotent stage [54]. Third, the present approach for generating OSKM-SLUG-driven iNPCs, acquiring highly proliferative transient intermediates, may allow large amounts of material to be produced for renal repair and regeneration as an alternative to whole kidney transplantation. The viability and properties of the generated iNPCs were maintained upon long-term sub-culturing and cryopreservation. In addition, progenitor cells are more desirable for transplantation due to their efficient engraftment and better integration into target tissues [54]. The potential “memory of origin” and lineage similarity of UC-derived iNPCs may mean that these cells have a better capacity to integrate into injured renal tissues, thereby improving their therapeutic effectiveness [55].

The present study demonstrated that human UC-derived iNPCs undergo long-term expansion in serum-free conditions. Prior to clinical trials, further studies are required for developing a non-integrating genetic delivery system that avoids potential issues associated with viral integration as well as their in vivo performance. In particular, the generated NPCs could be verified by further studies focused on engraftment, renal repair and regeneration, function, inflammation, fibrosis, and oxidative stress in acute and chronic mouse models of kidney diseases. Furthermore, the therapeutic potential of the iNPC secretome could be evaluated as a cell-free tool against nephrotoxicity. Nonetheless, this study proposes a novel method for generating expandable human iNPCs which may facilitate their broad applicability in a rapid, efficient, and patient-specific manner. In addition, these cells would be useful for pharmacological screening and as an in vitro platform to explore the cellular and molecular cues that govern kidney repair and regeneration.

## 4. Materials and Methods

### 4.1. Isolation and Culture of Human Urine Cells

Human UCs were obtained from two healthy donors (female and male of 36 and 34 years old, respectively), by modifying the isolation method as previously described [56]. This procedure was approved by the Institutional Review Board of Korea University (No. -1040548-KU-IRB-18-62-A-2). Briefly, 250 mL of fresh midstream urine samples were stored at 4 °C and, within 2 h, these samples were pelleted by centrifugation at 400× *g* for 10 min at room temperature. The resulting pellets were washed by resuspending in 10 mL PBS containing 1% penicillin/streptomycin/antimycotic solution (P/S/A, Hyclone, Logan, UT, USA), after centrifugation at 400× *g* for 10 min, resuspended and seeded in a gelatin-coated 12-plate (Sigma, St. Louis, MO, USA) and afterward in urine primary culture medium composed of DMEM/F12 (Hyclone, Logan, UT, USA) supplemented with 10% FBS (Hyclone, Logan, UT, USA), 1% P/S/A, and 2 mM L-glutamine. Then, 1 mL of fresh primary medium was added daily for 3 days and replaced by urine proliferation medium for the further culture of attached UCs (regarded as passage 1). Urine proliferation medium was a 1:1 ratio of high-glucose DMEM:REGM (Lonza, Basel, Switzerland) containing 5% FBS, 0.5% P/S, 2 mM L-glutamine, 1% non-essential amino acid solution (Thermo Fisher Scientific, Waltham, MA, USA), 2.5 ng/mL basic fibroblast growth factor (bFGF, PeproTech, Rocky Hill, NJ, USA), and 2.5 ng/mL epidermal growth factor (EGF, PeproTech, Rocky Hill, NJ, USA).

### 4.2. Generation of iNPCs

To induce reprogramming of human urine cells into iNPCs, human UCs were infected in various combinations of the retroviruses expressing SIX1, SIX2, OSR1, PAX2, OCT4, SOX2, KLF4, c-MYC, and SLUG. As previously established [57,58], these retroviruses were produced by the human 293-derived retroviral packing cell line transfected with pMXs-based retroviral vectors encoding hOCT3/4 (Plasmid #17217), hSOX2 (Plasmid #17218), hKLF4 (Plasmid #17219), hc-MYC (plasmid #17220) and SLUG using Lipofectamine 2000 (Life Technologies, Waltham, MA, USA), according to the manufacturer’s instructions. At 96 h post-transfection, retrovirus containing supernatants were harvested, filtered with a 0.45 μm sterile syringe filter (Millipore, Burlington, VT, USA), and concentrated at 20,000× *g* for 2 h at 4 °C. Human-derived UCs were seeded in a gelatin-coated 6-well plate and, 24 h later, exposed to the concentrated retrovirus in the presence of 4 μg/mL polybrene (Sigma, St. Louis, MO, USA). At 2 days after infection, UCs were re-seeded at a density of 5 × 10^4^ per well into a Matrigel-coated 6-well plate and incubated in UC culture medium for 2 days. Then, the infected UCs were exposed to a serum-free medium (mNPEM) composed of advanced RPMI 1640 (Gibco, Grand Island, NY, USA) supplemented with 100 ng/mL FGF9 (Peprotech, Rocky Hill, NJ, USA), 30 ng/mL BMP7 (Peprotech, Rocky Hill, NJ, USA), 1.25 μM CHIR 99021 (R&D Systems, Minneapolis, MN, USA), and antibiotics under a humidified incubator with 5% CO_2_ in air at 37 °C. iNPC-like colonies were observed within 12 days post-induction, the colonies were picked up, trypsinized to single cells, and plated onto Matrigel-coated plates in mNPEM. iNPCs were maintained in mNPEM and subcultured every 4–5 days. More details for forced expression of renal lineage-specific TFs in UCs were described in Supplementary method 1.

### 4.3. In Vitro Differentiation Potential of iNPCs

UC-derived NPCs were seeded in a fibronectin (Sigma, St. Louis, MO, USA)-coated plate and stabilized in mNPEM containing FGF9, BMP7, and CHIR99021 for 24 h. For podocyte differentiation, iNPCs were cultured in a VRAD medium composed of DMEM/F12 supplemented with 100 nM Vitamin D3 (Sigma-Aldrich, St. Louis, MO, USA), 60 mM RA, and 10% FBS for 7 days as previously described [34]. UCs and ESC-NPCs were differentiated for negative and positive controls in the same manner, respectively. The endocytic uptake of FITC-labeled albumin was determined as previously described [38,39]. Briefly, at day 7 of differentiation, the cells were serum-starved overnight, then were incubated with 1 mg/mL FITC-conjugated bovine serum albumin (Sigma-Aldrich, St. Louis, MO, USA) for 2 h at 4 °C and 37 °C. 4 °C was used as a control to prevent endocytosis. After that, cells were fixed with 4% paraformaldehyde (PFA) and stained with DAPI for nuclei. Images were acquired using an Olympus 1X81 inverted fluorescence microscope.

For proximal tubular cell differentiation, NPCs were cultured in DMEM/F12 supplemented with 1% insulin-transferrin-selenium (ITS) (Gibco, Grand Island, NY, USA), 10 ng/mL EGF, and 10% FBS for 21 days as previously described [32]. The medium was changed every two days. UCs and ESC- NPCs were differentiated with the same conditions as negative and positive controls, respectively. The ability of dextran uptake was used to evaluate the functional activity of renal proximal tubular cells [40]. Briefly, the differentiated cells at day 21 of differentiation were incubated with 10 μg/mL Alexa Fluor 555 dextran (Thermo Fisher Scientific) at 37 °C for 24 h. After that, cells were fixed with 4% PFA and stained with DAPI for nuclei count. Images were acquired using an Olympus 1X81 inverted fluorescence microscope.

In addition, to investigate whether the generated iNPCs could undergo nephrogenesis in vitro, they were further cultured by modifying the differentiation method as previously described [37]. iNPCs were seeding in a Matrigel-coated plate and stabilized in mNPEM for 24 h. The medium was changed to advanced RPMI (Gibco) supplemented with 1% L-glutamine, 1% P/S, 3 ng/mL CHIR, and 10 ng/mL FGF9 and, after 2 days, replaced and cultured by advanced RPMI supplemented with 1% L-glutamine, 1% P/S and 10 ng/mL FGF9 for 5 days. Afterward, the cells were exposed to an advanced medium supplemented with only 1% L-glutamine and 1% P/S for 5–7 days. The growth factors and small molecules used in the above experiments are shown in Table S1.

### 4.4. Quantitative RT-PCR (qRT-PCR) and RT-PCR

Total RNA was extracted using TRIzol reagent (Life Technologies, Carlsbad, CA, USA). Complementary DNA was synthesized using RT premix (Bioneer, Daejeon, South Korea) and oligo-dT (Life Technologies, Carlsbad, CA, USA), according to the manufacturer’s instructions. For quantitative RT-PCR, first-strand cDNA was generated using 500 ng of RNA, RT premix (Bioneer), oligo-dT (Life Technologies), SYBR Supermix (Bio-Rad, Hercules, CA, USA). Subsequent PCRs were performed in a final volume of 20 μL containing 1 μL of cDNA and 1 μL of 10 pM primers in PCR-Premix (Bioneer). PCR conditions were 95 °C for 10 min, followed by 40 cycles of 95 °C for 10 s, 58 °C for 50 s, and 72 °C for 20 s. Relative changes were analyzed using the 2-ΔΔCt method. The ratio of the mRNA level of the target to that of GAPDH was calculated. At least three independent samples were analyzed. The primer sequences used in this study are listed in Table S2.

### 4.5. Immunocytochemistry

Cells were fixed with 4% PFA for 20 min at RT, washed with PBS, and permeabilized with 0.03% Triton X-100 (Sigma, St. Louis, MO, USA) for 40 min at RT. Thereafter, cells were blocked with 3% bovine serum albumin and incubated with appropriate primary antibodies overnight at 4 °C. Finally, cells were washed three times with PBS and incubated with a secondary antibody (1:500) for 1 h at RT. The antibodies used in this study are listed in Table S3. Images were acquired using an Olympus 1X81 inverted fluorescence microscope.

### 4.6. Western Blot Analysis

For western blotting, cells were resuspended in ice-cold cell lysis buffer containing a protease inhibitor cocktail (Roche, Basel, Switzerland). Total proteins were extracted using RIPA buffer containing a protease inhibitor cocktail (Roche Molecular Diagnostics, Pleasanton, CA, USA). Lysates were centrifuged at 12,000× *g* for 30 min at 4 °C. Protein concentrations were determined using a Bradford assay kit (Bio-Rad, Hercules, CA, USA). Proteins were separated by SDS-PAGE on a 4–12% gradient-precast gel and transferred onto a polyvinylidene difluoride membrane (EMD Millipore, Burlington, MA, USA). The membranes were blocked in Tris-buffered saline containing 0.1% Tween 20 and 3% skim milk at RT for 1 h, incubated with primary antibodies overnight at 4 °C, and then labeled with horseradish peroxidase-conjugated IgG. Signals were visualized using SuperSignal West Pico Chemiluminescent Substrate (Thermo, Waltham, MA, USA). Kidney proteins were extracted with a protein extraction kit (Bio-Rad, Hercules, CA, USA). The protein samples (80 μg each) were separated by SDS-PAGE on an 8% gel and transferred onto a polyvinylidene difluoride membrane (Millipore, Bedford, MA, USA). The membranes were blocked in Tris-buffered saline containing 0.1% Tween 20 and 5% non-fat skim milk powder for 1 h and then incubated with primary antibodies overnight at 4 °C (Table S3). Bands were detected with horseradish peroxidase-conjugated secondary antibodies (1:1000, Abcam, Cambridge, MA, USA) and an enhanced chemiluminescence system (Pierce, Rockford, LA, USA).

### 4.7. RNA-Seq

Total RNA was extracted from UCs, iNPCs, and ESC-NPCs using TRIzol reagent (Thermo Fisher Scientific) according to the manufacture’s protocol. RNA quality was assessed by Agilent 2100 bioanalyzer using the RNA 6000 Nano Chip (Agilent Technologies, Amstelveen, The Netherlands), and RNA quantification was determined by using ND-2000 Spectrophotometer (Thermo Inc., DE, USA). Libraries were prepared from ~2 ng of total RNA from each sample by using the SMARTer Stranded RNA-Seq Kit (Clontech Laboratories, Inc., Mountain View, CA, USA). The isolation of mRNA was performed using the Poly(A) RNA Selection Kit (LEXOGEN, Inc., Wien, Austria), and the resulting mRNAs were used for the cDNA synthesis and shearing, according to the manufacturer’s instructions. Indexing was performed using the Illumina indexes 1–12. The enrichment step was carried out using PCR. Quantification was performed using the library quantification kit using a StepOne RealTime PCR System (Life Technologies, Inc., USA). High-throughput sequencing was performed as paired-end 100 sequencing using HiSeq 2500 (Illumina, Inc., San Diego, CA, USA). RNA-Seq reads were mapped using the Top Hat software tool (Toronto, ON, Canada) to obtain the alignment files, which were used for assembling transcripts, estimating their abundances, and detecting differential expression of genes or isoforms using cufflinks. FPKM (fragments per kilobase of exon per million fragments) was used to determine the expression level of the gene regions. Gene classification was based on searches performed with DAVID online tools (https://david.ncifcrf.gov/, accessed on 3 November 2021). The library preparation and RNA sequencing were performed as the NGS services provided by Ebiogen Inc. (Seoul, Korea). All raw data by RNA-seq data were deposited in the NCBI GEO database (accession number GSE134145).

### 4.8. Flow Cytometry

To examine the SIX2+ population, cells were harvested with 0.05% Trypsin/EDTA, rinsed three times with cold PBS, fixed with 0.5% PFA for 20 min, permeabilized with 0.1% Triton X-100, and stained with an anti-SIX2 antibody (1:250) followed by an Alexa Fluor 488-conjugated secondary antibody. All flow cytometric analyses were performed using a FACS Verse flow cytometer (BD Bioscience, San Jose, CA, USA).

### 4.9. Karyotyping

Karyotyping was performed by GTG banding, which was conducted by Samkwang Medical Laboratories (Seoul, Korea).

### 4.10. Re-Aggregation Assay with Mouse Embryonic Kidney Cells

The animal experiment was approved by the Institutional Animal Care & Use Committee at Korea University. Re-aggregation assay was performed as previously described [40,41,42]. Briefly, mouse kidney cells (mKCs) were isolated from E12.5 embryos. iNPCs and mKCs were mixed as a ratio of 1:10, a total number of 10^5^ cells was transferred into a low-binding PCR tube and centrifuged to form a single re-aggregate at 190× *g* for 5 min. The PCR tube with mixed cells were placed in a humidified incubator with 5% CO_2_ in air at 37 °C to allow aggregate formation with KCM (MEM supplemented with 10% FBS) supplemented with 10 µM ROCK inhibitor Y27632. The next day, the cell aggregates were transferred onto a 12-well Transwell plate (0.4-µm pore size, catalog #3460, Corning Costar, Corning, NY, USA) and cultured at the air-liquid interface of KCM for 7 days. After 7 days of culture, the resulting aggregates were fixed with 4% PFA and immunostained.

### 4.11. Statistical Analysis

All data are expressed as mean ± standard deviation (SD) for at least three replicates. Data comparisons were performed by paired two-tailed Student’s t and one-way ANOVA with Tukey’s post hoc tests for multiple comparisons. Differences with *p* < 0.05, 0.01, 0.001 were considered statistically significant.

## Data Availability

All data generated during this study are included in this article and its supplementary files. RNA-sequencing data have been deposited in NCBI’s Gene Expression Omnibus (GEO) with accession number GSE134145.

## Supplementary Materials

The following are available online at https://www.mdpi.com/article/10.3390/ijms222413449/s1.
