# Supplementary material for: Generation of Induced Nephron Progenitor-like Cells from Human Urine-Derived Cells
Source: Int J Mol Sci. 2021 Dec 15;22(24):13449. doi: 10.3390/ijms222413449 (PMC8708572; doi:10.3390/ijms222413449)
Supplement: Supplementary file 1 [file ijms-22-13449-s001.zip › ijms-1472430-supplementary.pdf]

# Supplemental Information

## Generation of Induced Nephron Progenitor-like Cells from Human Urine-Derived Cells

Wei-Wei Gao [1,2,†], Jie Zheng [1,3,†], Wonjin Yun [1], Phil-Jun Kang [1], Gyuman Park [1,2], Gwonhwa Song [1,3,*], In-Yong Kim [1,3,*], Seungkwon You [1,3,*]

**Affiliations**

[1] Department of Biotechnology, College of Life Sciences and Biotechnology, Korea University, Seoul 02841, Korea

[2] StemLab, Venture Incubation Center, Korea University, Seoul 136-701, Korea

[3] Institute of Animal Molecular Biotechnology, College of Life Sciences and Biotechnology, Korea University, Seoul 02841, Korea

* Correspondence: ghsong@korea.ac.kr (G.S.); iykim@korea.ac.kr (I.Y.K.); bioseung@korea.ac.kr (S.Y.)

† These authors contributed equally to this work.

# Contents


## Supplementary methods

**1. Forced expression of renal lineage-specific TFs in UCs**

**2. Maintenance and differentiation of hESCs**

**3. Tumorigenicity assay**

## Supplementary tables

**Table S1. Growth factors and small molecules used in this study**

**Table S2. Primer information for real-time PCR used in this study**

**Table S3. Primary antibodies used in this study**

## Supplementary figures

**Figure S1. Screening TF factors for iNPCs generation**

**Figure S2. Differentiation of Nephron progenitor cells from human ESCs**

**Figure S3. Biosafety study of iNPCs**

**Figure S4. *In vitro* propagation of human UCs derived -iNPCs**

**Figure S5. Expression of genes related to nephron development in UCs,**

**iNPCs and ESC-NPCs**

**Figure S6. Differentiation potential of iNPCs.**

**Figure S7. Formation of glomeruli and renal tubules from human ESC-NPCs**

## Supplementary methods

### 1. Forced expression of renal lineage-specific TFs in UCs

The human SLUG (SNAI2) fragment was excised from the pPGS-CMV-CITE-Neo (Plasmid 25696, Addgene) vector using the restriction enzyme Not1 and inserted into the Not1 site of the pMXs-puro back bone vector (Cell Biolab Inc., San Diego, CA, USA). The human OSR1 fragment was excised from the pCS2+MT (Addgene, #30491) vector using the restriction enzyme Xhol (Takara Bio Inc., Kusatsu, Japan) and SnaB1 (Takara Bio Inc., Kusatsu, Japan), this fragment was blunted using Klenow and inserted into the Not1 site of the pMXs-puro backbone vector (Cell Biolabs Inc., San Diego, CA, USA) that also with blunted treatment. The human PAX2 fragment was excised from the pCMV (Addgene, #36052) vector using the restriction enzyme Sall and Ascl, this fragment was blunted using Klenow and inserted into the Xhol site of the pMXs-puro backbone vector that also with blunted treatment. The human SIX1 fragment was excised from the MSCV (Addgene, #49263) vector using the restriction enzyme BamHI (Takara Bio Inc.) and NotI (Takara Bio Inc.), and inserted into the BamHI and NotI site of the pMXs-puro backbone vector. The fragment of SIX2 was excised from the pOTB7 (Korea human gene bank, hMU012822) vector using the restriction enzyme EcoR1 (Takara Bio Inc.) and NotI (Takara Bio Inc.), and inserted into the EcoR1and NotI site of pMXs-puro backbone vector. The constructed plasmids were confirmed by Enzyme cut. Retroviruses were produced by transfecting the human 293-dervied retroviral packing cell line with pMXs-based retroviral vectors encoding SLUG, OSR1, PAX2, SIX1 and SXI2 using Lipofectamine 2000 (Life Technologies, Waltham, MA, USA) according to the manufacturer's instructions. At 96h post-transfection, retrovirus containing supernatants were harvested, filtered with a 0.45 μm sterile syringe filter (Millipore, Bur-lington, VT, USA), and concentrated at 20000 g for 2h at 4 °C.

Human-derived UCs were seeded in a gelatin-coated 6-well plate and, 24 h later (about 30~50% confluent), exposed to the concentrated retrovirus (5TFs) in the presence of 4 μg/mL polybrene (Sigma, St. Louis, MO, USA). As previously reported [1], UCs infected with GFP retroviruses performed as control for monitoring infection efficiency by checking the expression of the pMXs-GFP virus with a fluorescence microscope. Two days after infection, more than 90% of the cells are GFP positive was observed, meanwhile UCs infected with GFP or 5TFs have reached 80-90% confluence. At this time 5TFs infected UCs were re-seeded at a density of $5\times10^4$ per well into a Matrigel-coated 6-well plate and incubated in UC culture medium for 2 days. Then, the infected UCs were exposed to a serum-free medium composed of advanced RPMI 1640 (Gibco，Grand Island, NY, USA) supplemented with 100 ng/ml FGF2 (Peprotech, Rocky Hill, NJ, USA), 100 ng/mL FGF9 (Peprotech, Rocky Hill, NJ, USA), 10 ng/ml activin A (Peprotech, Rocky Hill, NJ, USA), 1 μM retinoic acid (Sigma-Aldrich) and antibiotics under a humidified incubator with 5% CO2 in air at 37 °C [2]. iNPC-like colonies formation was examined within 9-15 days after induction, at day 15 post-induction, few putative iNPCs-like colonies were observed, these colonies were picked up, trypsinized into single cells and plated on Matrigel-coated plates in above inducting medium.

**2. Maintenance and differentiation of hESCs**

H9-ESCs (passages 55-65) and BG01-ESC (passages 50-65) were routinely maintained in a feeder-free culture system, cells were cultured on Matrigel coated 6 well-plate in E8 medium. Cultures were passage using Accutase (Sigma) at a 1:50 split ratio every 4-5days. We performed the differentiation protocol as previously described with some modification [3], for nephron

progenitor cells differentiation, cells were placed on Matrigel coated plate and fed with E8 medium until they reached 60-70% confluency, firstly cells were induced to late primitive streak cells by cultured in advanced RPMI supplemented with 8μM CHIR and 1% L-glutamine for 4 days. Then cells were cultured in advanced RPMI supplemented with 10ng/ml activin and 1% L-glutamine for 3 days to induced posterior intermediate mesoderm, finally cells were cultured in advanced RPMI supplemented with 10ng/ml FGF9 and 1% L-glutamine for 2 days to induce nephron progenitor cells. For *in vitro* tubulogenesis of hESCs derived NPCs, the medium was changed to advanced RPMI supplemented with 1% L-glutamine, 3ng/ml CHIR and 10ng/ml FGF9 for 2days, then medium was changed to advanced RPMI supplemented with 1% L-glutamine, 1% P/S and 10ng/ml FGF9 for 3days. After that, cells were cultured in advanced RPMI supplemented with 1% L-glutamine and 1% P/S for 7days.

**3. Tumorigenicity assay**

Animal experiment was approved by the Institutional Animal Care & Use Committee at the Korea University. A million of U-87MG, iPSCs, F-iNPCs, or M-iNPCs were resuspended individually in 200 μL of Matrigel solution (Corning, New York, USA) in the present of 10μM Y27632 and injected subcutaneously into the flank of BALB/c Nude mice. Afterwards, tumor formation was monitored over three months.

**Table S1. Growth factors and small molecules used in this study.**

| Growth factor/chemical | Company | Cat. No. |
|---|---|---|
| Activin A | Peprotech | 120-14 |
| LDN-193189 | Peprotech | 1062443 |
| CHIR99021 | R&D Systems | 4953/50 |
| FGF9 | Peprotech | 100-23 |
| FGF2 | Peprotech | 100-18B |
| EGF | Peprotech | 100-47 |
| BMP7 | Peprotech | 120-03 |
| Retinoic Acid, all trans | Sigma-Aldrich | R 2625 |
| Y27632 | Sigma-Aldrich | Y27632 |
| Heparin | Sigma-Aldrich | H3393 |
| Vitamin D3 | Sigma-Aldrich | PHR1237 |
| Insulin-transferrin-selenium (ITS) | Gibco | 41400-045 |

**Table S2. Primers used for RT-PCR and real-time PCR in this study.**

| Gene | Forward primer (5'-3') | Reverse primer (5'-3') |
|---|---|---|
| SIX2 | CTCAAGCACACTACATCGAG | GTTGTGGCTGTTAGAATTGGA |
| CITED1 | CAGCATCACTTCCCGCCAATTT | TTGCGATCTTTCACCGCAAGG |
| WT1 | TGTGTGCTTACCCAGGCTGCAA | CCGGGAGAACTTTCGCTGACAA |
| NCAM1 | CGATCTCATGGTTTCGGGATGG | TCATCAAACTGCACCTGGGCTG |
| GDNF | TTGGGTCTGGGCTATGAAACCAAG | GGTCATCATCAAAGGCGATGGGT |
| HOXD11 | ACAGGGCTTCGACCAGTTCTACGAG | TCAGTGAGGTTGAGCATCCGAGAGA |
| PAX2 | TTGAGTTTGAGAGGCGACACGG | TTGATGCTGCCGGTCTCGTAGT |
| hEYA1 | GCGCTGTGCAAACATCTCAAGC | ACTTCGGTGCCATTGGGAGTCA |
| AQP1 | ATGCCGACGACATCAACCAG | TGAGTCGGTGAGCAACTTTGGG |
| CD13 | CCATGAAGGCCGAGTTCAACA | ATGAAGGCCAGCAAGTACGTG |
| E-cadherin | TGTTTGACTATGAAGGAAGCGG | CATTTCCCAGCACATGGGT |
| Podocalyxin | CTTGAGACACAGACACAGAG | CCGTATGCCGCACTTATC |
| Nephrin | TGGCTCGGACCAAACCAACATT | AGGGCCTCATACCTGATGCAGA |
| Synaptopodin | CGCTCACCACACCAACTTCTAA | CTAGAAAGTGGCAGGCTCTGTG |
| NANOG | ATAGCAATGGTGTGACGCAG | GATTGTTCCAGGATTGGGTG |
| OCT4 | GACAGGGGGAGGGGAGGAGCTAGG | CTTCCCTCCAACCAGTTGCCCCAAAC |
| GAPDH | GTGGTCTCCTCTGACTTCAACA | CTCTTCCTCTTGTGCTCTTGCT |

**Table S3. Primary antibodies used in this study.**

| Antibody | Host | Supplier | Dilution | Catalog number |
|---|---|---|---|---|
| Anti-SIX2 | Rabbit | Proteintech | 1: 250 | 11562-1-AP |
| Anti-CITED1 | Mouse | Abnova | 1:200 | H00004435 |
| Anti-E-CADHERIN | Mouse | BD Biosciences | 1:250 | 610181 |
| Anti-Lotus tetragonolobus lectin (LTL) | Biotin-conjugated | Vector Labs | 1:250 | B-1325 |
| Anti-AQP1 | Mouse | Santa Cruz | 1:100 | SC32737 |
| Anti-Podocalyxin (PODXL) | Goat | R&D Systems | 1:500 | AF1658 |
| Anti-SYNAPTOPODIN | Goat | Santa Cruz | 1:100 | SC21537 |
| Anti-E-cadherin | Goat | R&D | 1:200 | AF 648 |
| Anti-Human Nuclei | Mouse | Millipore | 1:250 | MAB 1281 |
| Anti-NEPHRIN | Rabbit | Abcam | 1:250 | Ab216341 |
| Anti-CD13 | Mouse | Abcam | 1:250 | Ab7414 |

S1.

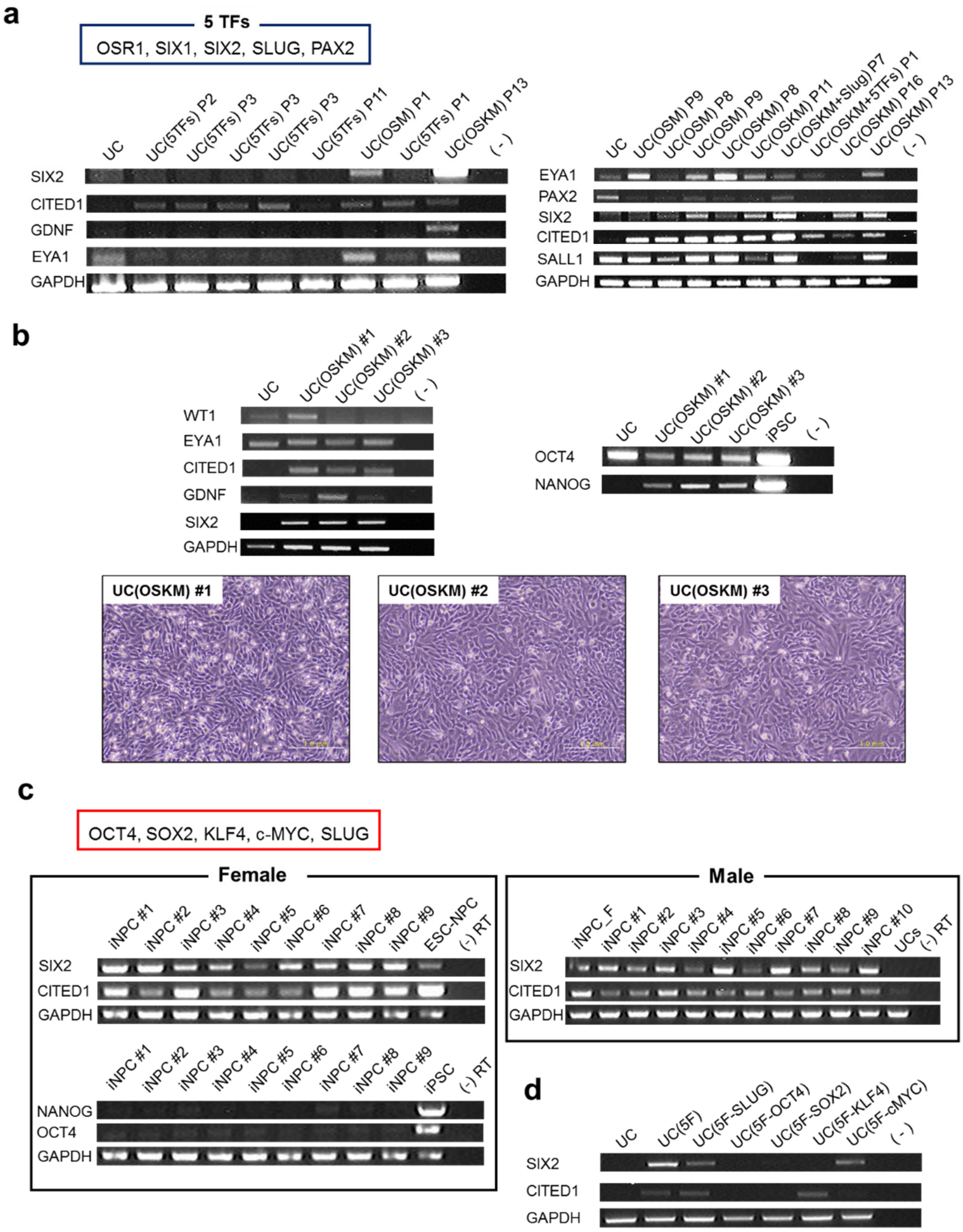


**Figure S1. Screening TF factors for iNPCs generation. a**, RT-PCR analysis of NPC markers in combinations of 5TFs, OSK, OSKM, OSKM-SL-infected UC, the expression of each gene was analyzed at in vitro expansion one passage. 5TFs:OSR1, SIX1, SIX2, SLUG, PAX2. O, OCT4; S, SOX2; K, KLF4; M, cMYc. **b**, RT-PCR gene expression analysis and morphology of 3 lines of UCs infected with OSKM TFs. NPC marker: WT1, EYA1, CITED1, GDNF, SIX2; pluripotent makers: NANOG, OCT4. **c**, RT-PCR analysis of NPC-specific and pluripotent markers in 5F TF-infected female UCs (left) and male UCs (right). NPC markers: SIX2 and CITED1, serving as a strong indicator for NPCs; pluripotent makers: NANOG, OCT4. **d**, RT-PCR analysis of NPC markers SIX2 and CITED1 in UCs infected with 5F, 5F-SLUG, 5F-OCT4, 5F-SOX2, 5F-KLF4, and 5F-cMYC at 12 days post-induction.

## S2.

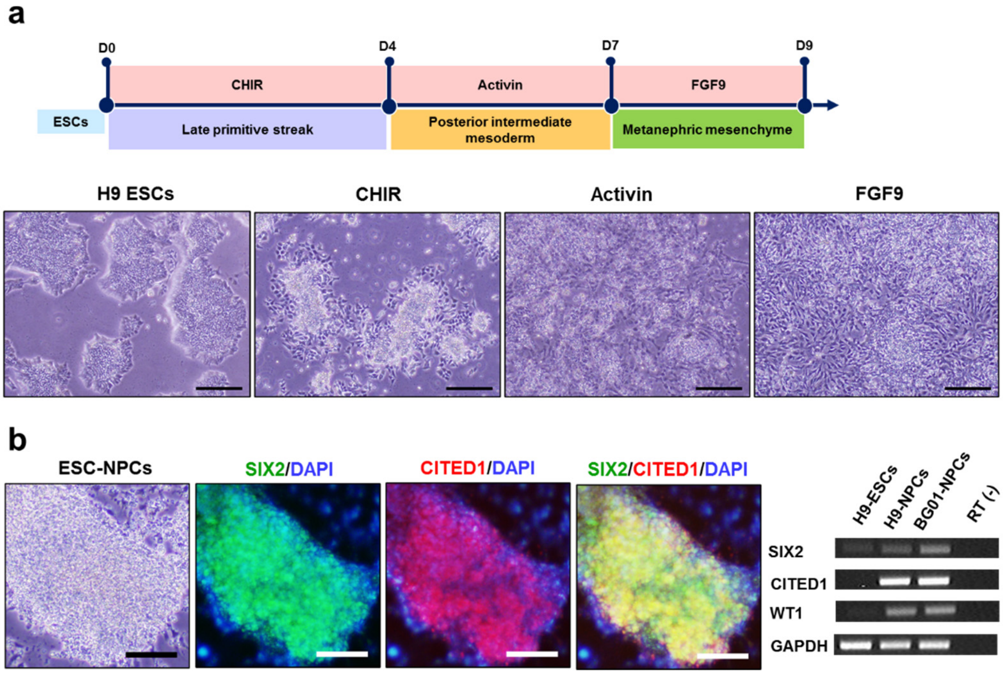


**Figure S2. Differentiation of NPCs from human ESC. a**, Schematic for differentiation of NPCs from human ESCs (top). Morphological change of hESCs-H9 during the differentiation process into NPC (bottom). **b**, Immunofluorescence staining (left) of SIX2 (green) and CITED1 (red) in human ESC derived-NPC and RT-PCR analysis of SIX2, CITED and WT1 in human ESC derived-NPC (right). Nuclei were counterstained with DAPI. Scale bars = 200 µm.

## S3.

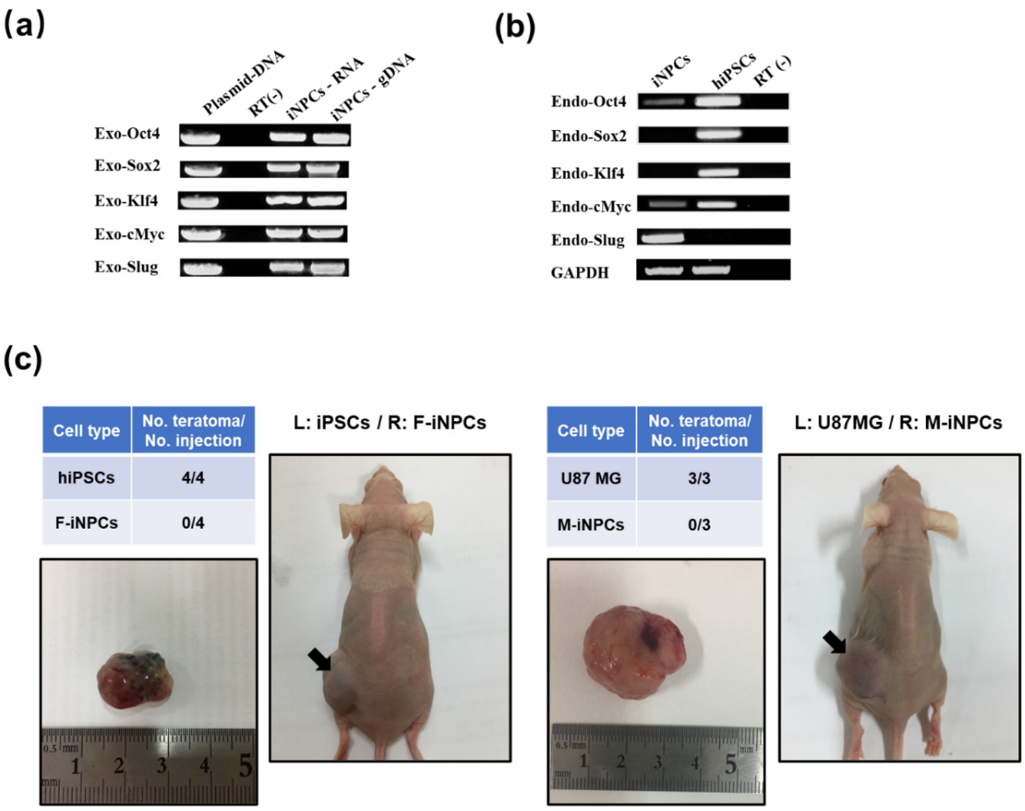


**Figure S3. Biosafety study of iNPCs. a**,**b**, Evaluation of exogenous and endogenous expression of each of the reprogramming genes in iNPC at passage 8, as demonstrated by RT-PCR. **c**, Tumorigenicity assay in nude mice upon injecting 1 × $10^6$ iPSCs or U87 MG cells in left dorsal flank and 1 × $10^6$ iNPC-F or iNPC-M in right dorsal flank. Block arrows indicate tumor formation in iPSCs or U87 MG cells injected site. Bottom images show the size and morphology of the tumor formed from iPSCs or U87 cells injected site, respectively.

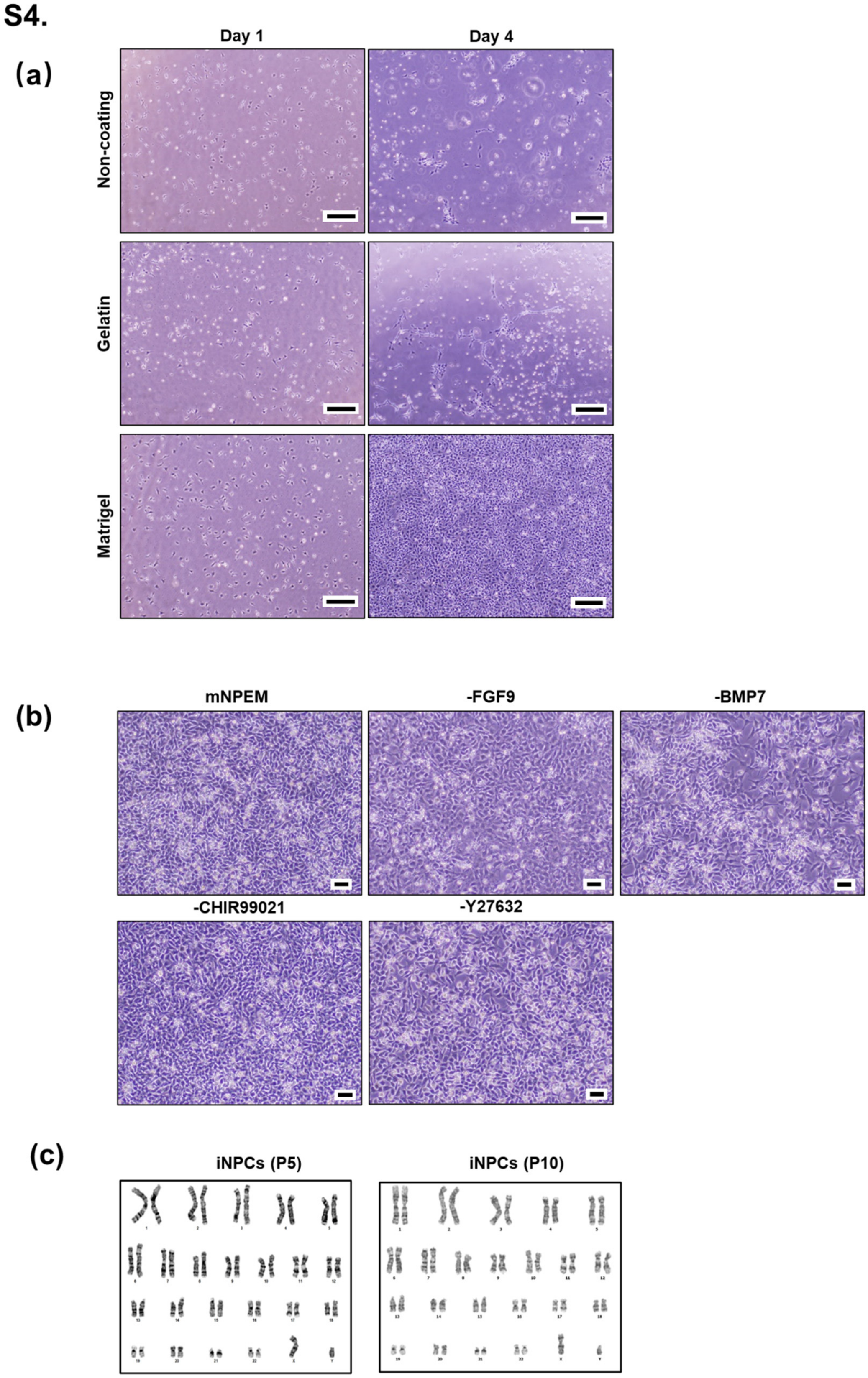


**Figure S4.** ***In vitro*** **expansion of human UC-derived iNPCs. a,b**, Optimization of culture conditions for In vitro expansion of iNPCs. **a**, Cell morphological changes of iNPCs in non-, gelatin- and Matigel-coated plates in mNPEM for 4 days. Cells were seeded at a density of $5 \times 10^4$ cells per well in a 6-well plate. **b**, Cell optical images at day 6 of iNPCs in Matigel-coated plates in the medium supplemented

with FGF9, BMP7, CHIR99021 and Y-27632, in combination or as individually removed. Cells were seeded at a density of 5 × $10^4$ cells per well in a 6-well plate. **c**, Karyotyping (G-banded) of iNPCs derived from male human UCs at passages 5 and 10.

**S5.**

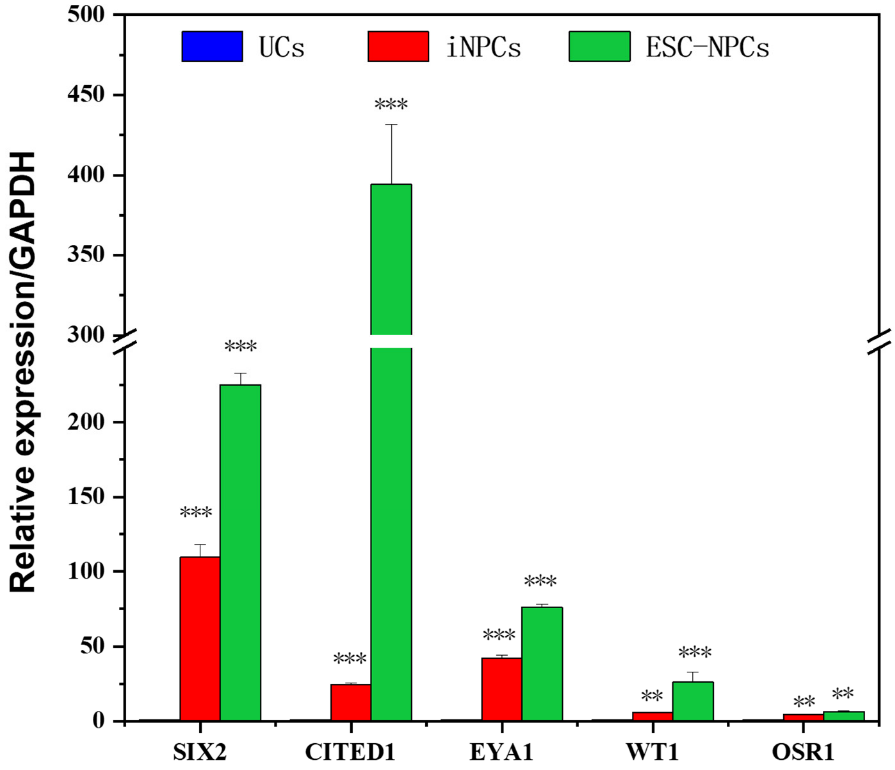


**Figure S5. Expression of genes related to nephron development in UCs, iNPCs and ESC-NPCs**. qRT-PCR analysis of nephron development related genes (SIX2, CITED1, EYA1, WT1 and OSR1) in UCs, iNPCs and ESC-NPCs. Data are represented as mean ± SD. * denotes a statistically significant difference when compared with UCs. ** $p < 0.01$, *** $p < 0.001$.

**S6.**

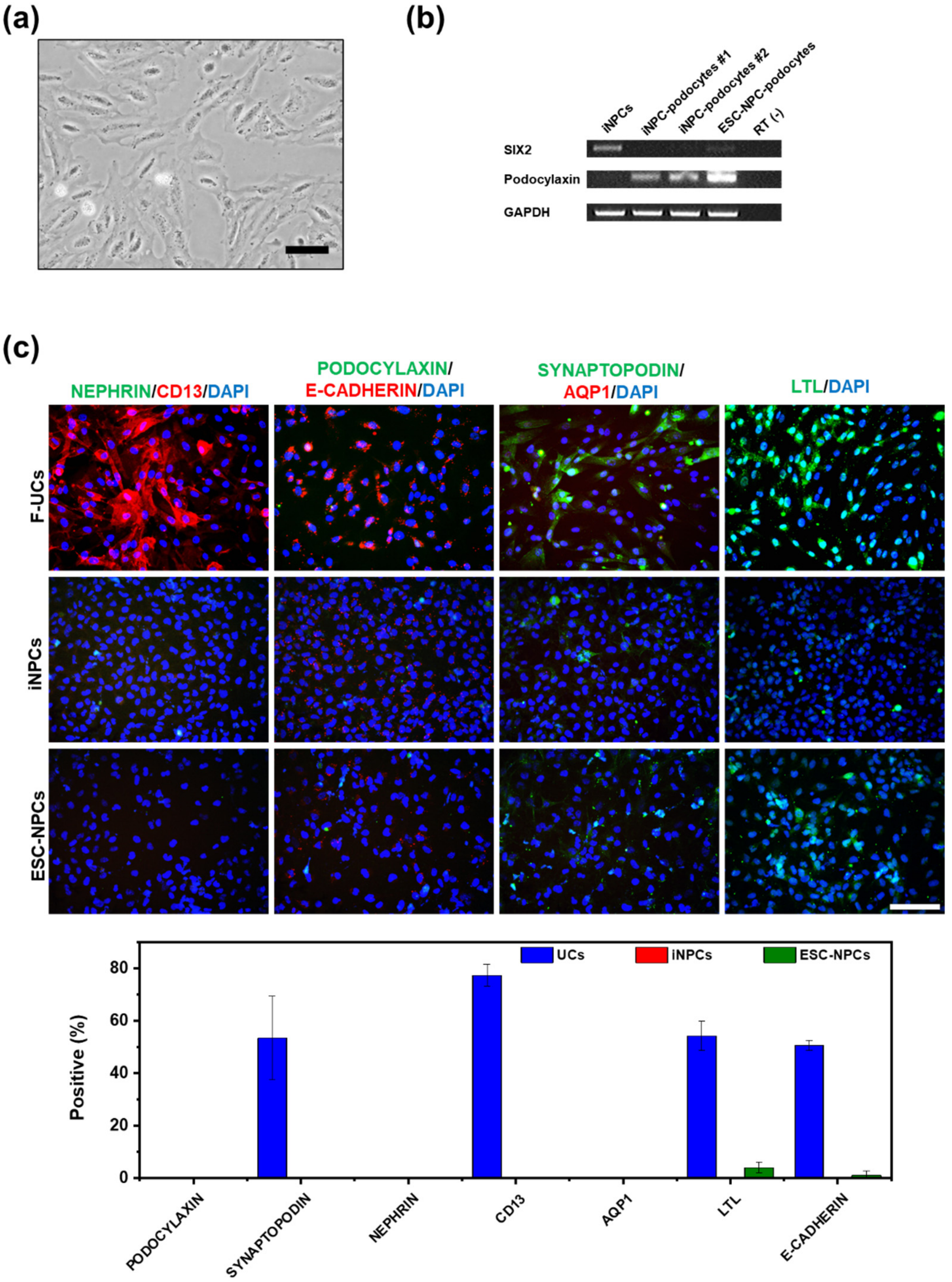


**Figure S6.** *Cont.*

**S6.**

(d)

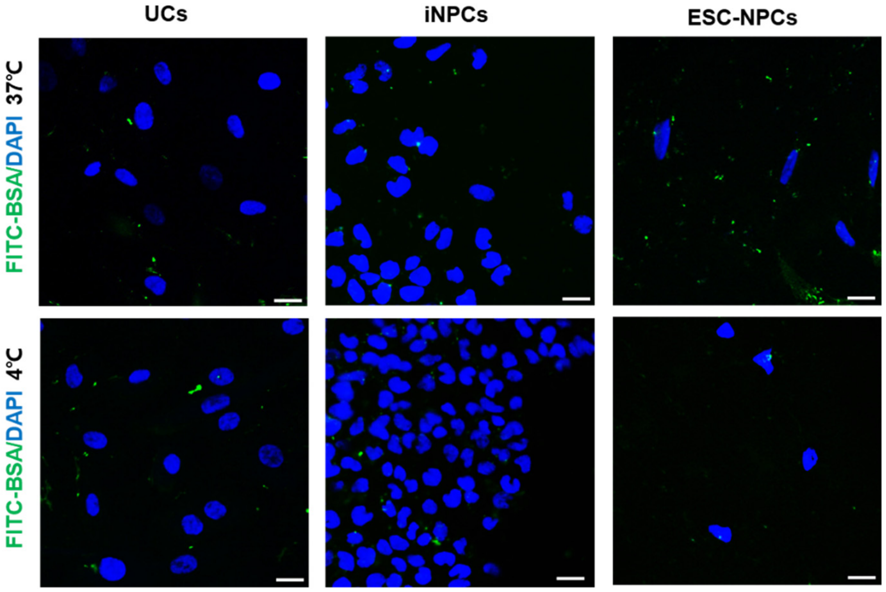


(e)

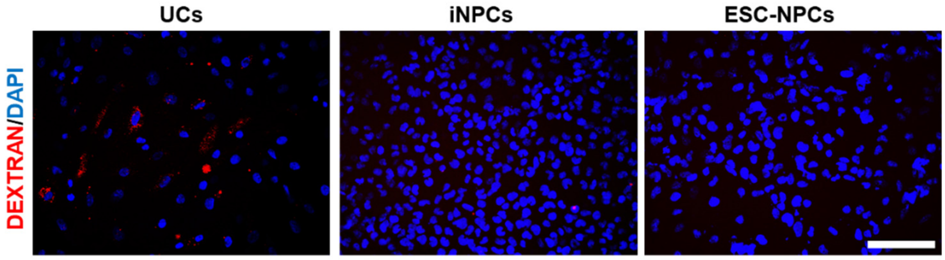


**Figure S6. Differentiation potential of iNPCs. a**, Optical images showing the morphology of podocytes induced from iNPCs. Scale bar =100 µm. **b**, RT-PCR analysis of NPC-specific marker SIX2 and podocyte-specific marker podocylaxin in iNPCs and ESC-NPC-podocytes. **c**, Immunofluorescence and Quantitative analysis of podocyte-specific markers of NEPHRIN (green), PODXL (green), and SYNAPTOPODIN (green) and renal tubular cell-specific markers CD13 (red), E-CADHERIN (red), AQP1 (red) and LTL (green) in UC, iNPCs and ESC-NPCs. Scale bar = 100 µm. **d**, Albumin uptake by UCs, iNPCs and ESC-NPCs before performing podocyte differentiation. Scale bar = 20 µm. **e**, Dextran uptake by UCs, iNPCs and ESC-NPCs before performing tubular differentiation. Scale bar = 100 µm.

## S7.

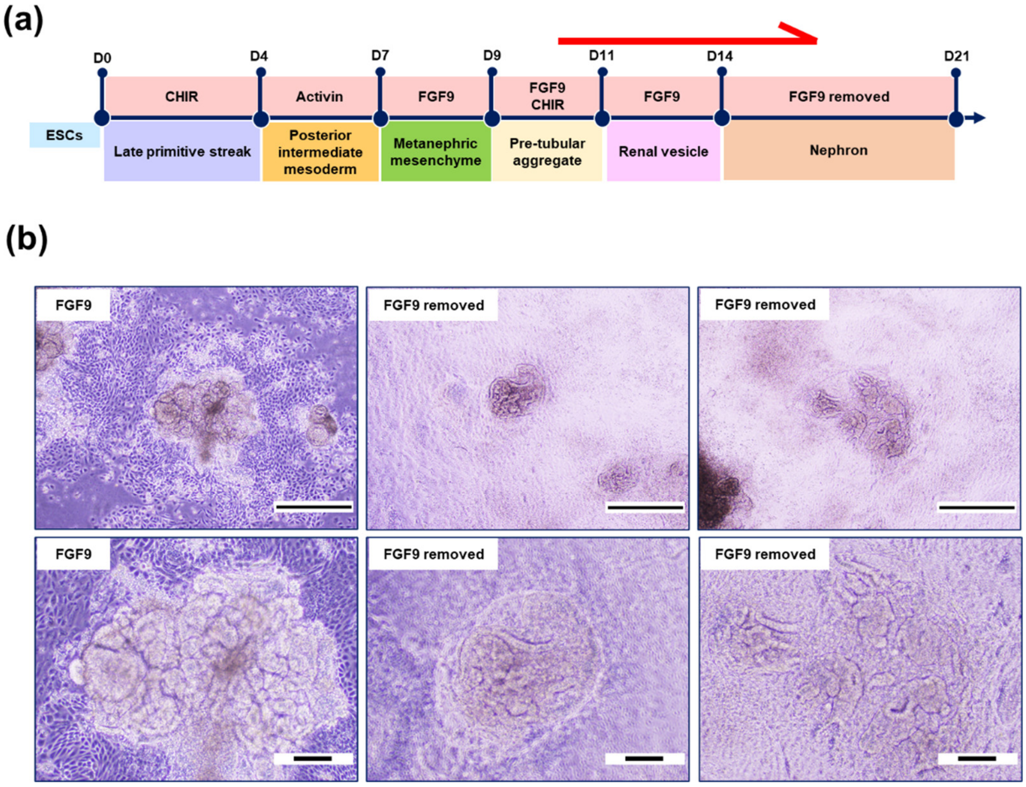


**Figure S7. Formation of glomeruli and renal tubules from human ESC-NPCs. a,b**, Schematic overview and optical images for formation of glomeruli and renal tubules from human H9 ESC. Scale bar = 1 mm (top) and 200 μm (bottom).

## References


1. Zhou, T.; Benda, C.; Dunzinger, S.; Huang, Y.; Ho, J. C.; Yang, J.; Wang, Y.; Zhang, Y.; Zhuang, Q.; Li, Y.; Bao, X.; Tse, H. F.; Grillari, J.; Grillari-Voglauer, R.; Pei, D.; Esteban, M. A., Generation of human induced pluripotent stem cells from urine samples. Nature protocols 2012, 7, (12), 2080-9.
2. Lam, A. Q.; Freedman, B. S.; Morizane, R.; Lerou, P. H.; Valerius, M. T.; Bonventre, J. V., Rapid and efficient differentiation of human pluripotent stem cells into intermediate mesoderm that forms tubules expressing kidney proximal tubular markers. Journal of the American Society of Nephrology : JASN 2014, 25, (6), 1211-25.
3. Morizane, R.; Lam, A. Q.; Freedman, B. S.; Kishi, S.; Valerius, M. T.; Bonventre, J. V., Nephron organoids derived from human pluripotent stem cells model kidney development and injury. Nature biotechnology 2015, 33, (11), 1193-200.
